# Biomarkers of the transsulfuration pathway and risk of renal cell carcinoma in the European Prospective Investigation into Cancer and Nutrition (EPIC) study

**DOI:** 10.1002/ijc.34009

**Published:** 2022-04-16

**Authors:** Joanna L. Clasen, Alicia K. Heath, Heleen Van Puyvelde, Inge Huybrechts, Jin Young Park, Pietro Ferrari, Ghislaine Scelo, Arve Ulvik, Øivind Midttun, Per Magne Ueland, Kim Overvad, Anne Kirstine Eriksen, Anne Tjønneland, Rudolf Kaaks, Verena Katzke, Matthias B. Schulze, Domenico Palli, Claudia Agnoli, Paolo Chiodini, Rosario Tumino, Carlotta Sacerdote, Raul Zamora‐Ros, Miguel Rodriguez‐Barranco, Carmen Santiuste, Eva Ardanaz, Pilar Amiano, Julie A. Schmidt, Elisabete Weiderpass, Marc Gunter, Elio Riboli, Amanda J. Cross, Mattias Johansson, David C. Muller

**Affiliations:** ^1^ Department of Epidemiology and Biostatistics School of Public Health, Imperial College London London UK; ^2^ International Agency for Research on Cancer Lyon France; ^3^ Department of Public Health and Primary Care, Faculty of Medicine and Health Sciences Ghent University Ghent Belgium; ^4^ Cancer Epidemiology Unit University of Turin Turin Italy; ^5^ Bevital A/S Bergen Norway; ^6^ Department of Public Health Aarhus University Aarhus C Denmark; ^7^ Danish Cancer Society Research Center Diet, Genes and Environment Copenhagen Denmark; ^8^ Division of Cancer Epidemiology German Cancer research Center (DKFZ) Heidelberg Germany; ^9^ Department of Molecular Epidemiology German Institute of Human Nutrition Potsdam‐Rehbruecke Nuthetal Germany; ^10^ Institute of Nutritional Science, University of Potsdam Nuthetal Germany; ^11^ Cancer Risk Factors and Life‐Style Epidemiology Unit Institute for Cancer Research, Prevention and Clinical Network—ISPRO Florence Italy; ^12^ Epidemiology and Prevention Unit, Department of Research Fondazione IRCCS Istituto Nazionale dei Tumori Via Venezian Milan Italy; ^13^ Dipartimento di Salute Mentale e Fisica e Medicina Preventiva, Università degli Studi della Campania ‘Luigi Vanvitelli’ Naples Italy; ^14^ Hyblean Association for Epidemiological Research (AIRE‐ONLUS) Ragusa Italy; ^15^ Unit of Cancer Epidemiology Città della Salute e della Scienza University‐Hospital Turin Italy; ^16^ Unit of Nutrition and Cancer, Cancer Epidemiology Research Programme, Catalan Institute of Oncology, Bellvitge Biomedical Research Institute (IDIBELL) Barcelona Spain; ^17^ Escuela Andaluza de Salud Pública (EASP) Granada Spain; ^18^ Instituto de Investigación Biosanitaria ibs.GRANADA Granada Spain; ^19^ Centro de Investigación Biomédica en Red de Epidemiología y Salud Pública (CIBERESP) Madrid Spain; ^20^ Department of Epidemiology Murcia Regional Health Council, IMIB‐Arrixaca Murcia Spain; ^21^ Navarra Public Health Institute Pamplona Spain; ^22^ IdiSNA, Navarra Institute for Health Research Pamplona Spain; ^23^ Ministry of Health of the Basque Government Sub Directorate for Public Health and Addictions of Gipuzkoa San Sebastian Spain; ^24^ Biodonostia Health Research Institute Epidemiology of Chronic and Communicable Diseases Group San Sebastián Spain; ^25^ Cancer Epidemiology Unit, Nuffield Department of Population Health University of Oxford Oxford UK; ^26^ Department of Epidemiology and Biostatistics, School of Public Health MRC‐PHE Centre for Environment and Health, Imperial College London London UK

**Keywords:** dietary biomarkers, kidney cancer, transsulfuration, vitamin B6

## Abstract

Previous studies have suggested that components of one‐carbon metabolism, particularly circulating vitamin B6, have an etiological role in renal cell carcinoma (RCC). Vitamin B6 is a cofactor in the transsulfuration pathway. We sought to holistically investigate the role of the transsulfuration pathway in RCC risk. We conducted a nested case‐control study (455 RCC cases and 455 matched controls) within the European Prospective Investigation into Cancer and Nutrition (EPIC) study. Plasma samples from the baseline visit were analyzed for metabolites of the transsulfuration pathway, including pyridoxal 5′‐phosphate (PLP, the biologically active form of vitamin B6), homocysteine, serine, cystathionine, and cysteine, in addition to folate. Bayesian conditional logistic regression was used to estimate associations of metabolites with RCC risk as well as interactions with established RCC risk factors. Circulating PLP and cysteine were inversely associated with RCC risk, and these associations were not attenuated after adjustment for other transsulfuration metabolites (odds ratio (OR) and 90% credible interval (CrI) per 1 SD increase in log concentration: 0.76 [0.66, 0.87]; 0.81 [0.66, 0.96], respectively). A comparison of joint metabolite profiles suggested substantially greater RCC risk for the profile representative of low overall transsulfuration function compared to high function (OR 2.70 [90% CrI 1.26, 5.70]). We found some statistical evidence of interactions of cysteine with body mass index, and PLP and homocysteine with smoking status, on their associations with RCC risk. In conclusion, we found evidence suggesting that the transsulfuration pathway may play a role in metabolic dysregulation leading to RCC development.

## Graphical abstract

What's new?

One‐carbon metabolism, owing largely to its relationship with DNA methylation, is implicated in cancer etiology. Whether alterations in the transsulfuration pathway—a critical unit of one‐carbon metabolism that is essential to homocysteine catabolism—has a role in cancer development, however, remains unknown. In our study, analysis of data from the European Prospective Investigation into Cancer and Nutrition cohort revealed an association between transsulfuration and renal cell carcinoma (RCC) risk. The association extends beyond the previously described role of vitamin B6 in RCC development and was characterized primarily by a marked increase in RCC risk relative to low overall transsulfuration function.

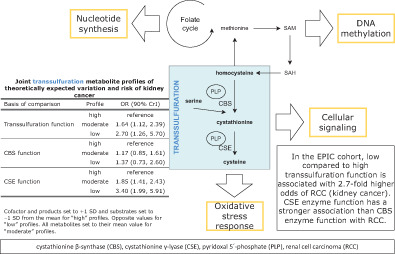

**Abbreviations**
BMI: body mass index
CBS: cystathionine β‐synthase
CrI: credible interval
CSE: cystathionine γ‐lyase
ELPD: expected log predictive density
EPIC: European Prospective Investigation into Cancer and Nutrition
OR: odds ratio
PLP: pyridoxal 5′‐phosphate
RCC: renal cell carcinoma
SAM: S‐adenosylmethionine
SDMA: symmetric dimethylarginine

## 1 INTRODUCTION

The incidence of kidney cancer is increasing, and it is currently the 14th most common cancer globally.^1^ Incidence is highest in developed countries, and twice as high among men compared to women.^1^ Around 90% of kidney cancer diagnoses are renal cell carcinoma (RCC).^2^ Established modifiable risk factors for RCC include overweight and obesity, cigarette smoking and high blood pressure.^3^ There is limited and inconclusive evidence on associations with dietary or nutrient factors. However, of particular interest is a previous finding of an association between increased circulating vitamin B6 concentration (measured in its biologically active form of pyridoxal 5′‐phosphate [PLP]) and a lower risk of RCC, with a 60% lower risk in the highest fourth vs the lowest fourth of plasma concentration.^4^ The possibility of shared causal pathways in RCC between the established risk factors and metabolic markers such as vitamin B6 has not been thoroughly examined.

PLP is a coenzyme in over 160 different reactions in multiple metabolic pathways.^5^ The inverse association between PLP and RCC risk may be explained by its role in one or more of these pathways, and other metabolic components in these pathways may be mechanistically related to RCC risk as well. The transsulfuration pathway, which requires PLP, is part of one‐carbon metabolism along with the folate and methionine cycles. In the methionine cycle, homocysteine can either continue the cycle and be remethylated into methionine, which is converted to S‐adenosylmethionine (SAM), or it can exit the methionine cycle and be irreversibly catabolized by the transsulfuration pathway into cysteine.^6^ PLP is a coenzyme both for the enzyme cystathionine β‐synthase (CBS), which condenses homocysteine and serine to create cystathionine, and for the enzyme cystathionine γ‐lyase (CSE), which then converts cystathionine to cysteine^7^ (Figure S1). SAM is an activator of CBS, and folate indirectly influences CBS activity because of its robust linear positive association with SAM.^8^ RCC is of particular interest because the kidney, along with the liver, are where most amino acid metabolism occurs, including transsulfuration.^6^

The transsulfuration pathway is important for cell signaling and protein synthesis and is therefore critical in cell proliferation.^7^ Its downstream metabolite, glutathione, is important for the oxidative stress response, and cysteine is the limiting component in glutathione synthesis.^9^ Hydrogen sulfide, a gas‐signaling molecule with physiological effects associated with aging processes, is produced by transsulfuration enzymes.^10^ As part of the broader one‐carbon metabolism, transsulfuration is related to regulation of methylation reactions including DNA methylation, and nucleotide synthesis.^6^ Therefore, there are a variety of possible biological mechanisms that may link altered transsulfuration activity to cancer risk.

While the closely related methionine and folate cycles have been more heavily probed regarding their associations with cancer development, there is little evidence on the role of transsulfuration. In particular, because of the interdependent nature of biomarkers from a single metabolic pathway, we aimed to build upon exiting evidence, which has mostly focused on individual metabolites, with a holistic view of pathway‐wide associations. Using data from the same study that previously examined PLP and RCC risk,^4^ we extended the investigation to include additional components of the transsulfuration pathway in relation to RCC risk and assessed how these associations vary after adjustment for, and interaction with, established risk factors.

## 2 MATERIALS AND METHODS

### 2.1 Participants and data collection

The European Prospective Investigation into Cancer and Nutrition (EPIC) study is a multinational observational cohort consisting of over 500 000 participants recruited between 1992 and 2000. Participants provided blood samples, completed questionnaires on diet and lifestyle, and had anthropometric measurements taken. Details of recruitment and data collection have been published previously.^11^, ^12^

A nested case‐control study within EPIC was established after follow‐up through 2004 to 2010, depending on the center, with 556 histologically confirmed RCC cases and 556 controls matched on country, sex, date of blood collection (±1 month) and date of birth (±1 year). Controls were selected from the pool of all cohort members alive and without a reported cancer diagnosis at the time of the RCC case diagnosis. Exclusion criteria were a previous diagnosis of cancer other than nonmelanoma skin cancer, or a diagnosis of RCC that was not histologically confirmed (censored at the date of diagnosis).^4^ The Malmö, Sweden center did not participate.

Blood fractions were divided into aliquots in 0.5‐mL straws, which were heat sealed and stored in liquid nitrogen tanks at −196°C, except in Denmark, where samples were stored in 1‐mL tubes between −120 and −160°C.^12^ Biochemical analyses were performed at the Bevital A/S laboratory in Bergen, Norway. Plasma samples were analyzed for concentrations of PLP, homocysteine, serine, cystathionine, cysteine and folate. In addition, symmetric dimethylarginine (SDMA) and neopterin were measured as markers of kidney function and cellular immune activation, respectively. Total homocysteine, serine, cystathionine and total cysteine were measured with GC‐MS^13^, ^14^; PLP, SDMA and neopterin were measured with LC‐MS/MS^15^, ^16^; and folate was measured with a microbiological assay.^17^ Samples from each case with their matched control were analyzed together in the same batch, and laboratory staff were blinded to the case‐control status of all samples. Full details of the nested case‐control study have been previously published.^4^

### 2.2 Statistical analysis

Participants with missing data were excluded from analyses. Greece was excluded, and all participants from Norway were excluded due to missing data for anthropometric measurements. Baseline characteristics of cases and controls were compared using frequencies for categorical variables and the median and interquartile range for continuous variables. Correlations between transsulfuration metabolites were estimated in the controls using the Pearson correlation coefficient. Metabolite concentrations were log base 2 transformed, centered and scaled to an SD of one prior to their inclusion in regression analyses.

Multivariable Bayesian conditional logistic regression was used to estimate odds ratios (OR) and 90% credible intervals (90% CrI) for the associations of the transsulfuration metabolites with risk of RCC. Minimally adjusted models included a single metabolite, highest level of education (none or primary school, secondary school, technical/professional, university degree) and fasting status at the time of blood draw (<3 hours [not fasting], 3‐6 hours [in between], >6 hours [fasting]). Country, sex, age and date of blood draw were accounted for as matching factors. Further adjusted models for single metabolites additionally included body mass index (BMI, continuous) and smoking status (never, former, current). The full transsulfuration model included all five metabolites (PLP, homocysteine, serine, cystathionine and cysteine), and was adjusted for folate concentration, highest level of education, fasting status, BMI and smoking status. For the subset of participants with measured blood pressure data available, additional models were further adjusted for systolic blood pressure, diastolic blood pressure and both. All regression model results for continuous predictors are presented per 1 SD increase.

Because components of tightly regulated metabolic systems vary in concert with each other, and not in isolation, we were interested in the associations of pathway‐wide metabolite profiles with RCC risk, and so we compared joint risk estimates across multiple metabolites. These associations do not represent variation in a single metabolite while holding all others constant, but rather, represent patterns of variation across multiple related metabolites. We first defined four different types of profiles, each indicative of empirically or theoretically plausible variations of metabolites. Each set included three profiles, with the “mid” profile setting all metabolite values at their mean, and “low” and “high” profiles with equal magnitude distance from the mean in opposite directions. For example, where PLP is set to 1 SD below the mean for a low profile, it is 1 SD above the mean in the high profile. We used this approach to estimate associations with RCC risk for one set of empirically motivated profiles (PLP values were assigned then other metabolites were set to their conditional expected values from unadjusted linear regressions of the log concentration on log PLP) and three sets of theoretically determined profiles (regulatory control of overall transsulfuration, CBS and CSE function). These three sets were designed following the assumptions that a higher concentration of an enzymatic cofactor reduces the regulatory restriction of activity, which subsequently results in lower substrate concentrations and higher product concentrations. Therefore, high, mid and low overall transsulfuration function profiles had values set to 1, 0 and −1 respectively for PLP (cofactor) and cysteine (product) and −1, 0 and 1 for homocysteine and serine (substrates), while cystathionine did not vary because it is an intermediate. Likewise, the high, mid, and low CBS profiles had values set to 1, 0 and −1 for PLP and cystathionine, and −1, 0 and 1 for homocysteine and serine. The high, mid and low CSE profiles had values to 1, 0 and −1 for PLP and cysteine, and −1, 0 and 1 for cystathionine. Looking at separate sets of profiles for CBS and CSE may be informative because these two enzymes are controlled by different regulatory processes.

All analyses were conducted in R version 4.0.5 and Bayesian inference was done with the package RStanArm version 2.21.1.^18^, ^19^

## 3 RESULTS

This analysis included 455 RCC cases and 455 matched controls with complete data on all markers and covariates. A comparison of the included 910 participants vs 194 excluded with missing data is shown in Table S1. Among included participants, median age at recruitment was 57 years, median time from blood draw to case diagnosis was 7 years, and median age of cases at diagnosis was 64 years. The majority (56%) of pairs were men. Median BMI for cases and controls was 27.1 and 26.2 kg/m^2^, respectively. 32% of cases and 24% of controls were current smokers (Table 1). Median PLP for cases and controls was 30.4 and 36.4 nmol/L, respectively. All other metabolite concentrations were similar between cases and controls (Table 2). The strongest correlation between transsulfuration biomarkers in controls was for homocysteine and cysteine (*r* = 0.44) (Figure S2).

**TABLE 1:** Characteristics of RCC cases and matched controls in a nested case‐control study within EPIC

	Cases (N = 455)	Controls (N = 455)
Country		
Denmark	109 (24%)	109 (24%)
France	8 (2%)	8 (2%)
Germany	118 (26%)	118 (26%)
Italy	86 (19%)	86 (19%)
Spain	51 (11%)	51 (11%)
The Netherlands	43 (9%)	43 (9%)
United Kingdom	40 (9%)	40 (9%)
Sex		
Male	256 (56%)	256 (56%)
Female	199 (44%)	199 (44%)
Smoking status		
Never	172 (38%)	199 (44%)
Former	138 (30%)	148 (33%)
Current	145 (32%)	108 (24%)
Educational attainment		
Primary school or less	190 (42%)	173 (38%)
Secondary school	65 (14%)	59 (13%)
Technical/professional school	107 (24%)	111 (24%)
Longer education (incl. University deg.)	93 (20%)	112 (25%)
Fasting status		
Yes	130 (29%)	132 (29%)
In between	86 (19%)	85 (19%)
No	239 (53%)	238 (52%)
Hypertension		
No	232 (51%)	265 (58%)
Yes	157 (35%)	119 (26%)
Do not know	14 (3%)	15 (3%)
Missing	52 (11%)	56 (12%)
Systolic BP (mm Hg)^a^	138 (126, 152)	132 (120, 147)
Diastolic BP (mm Hg)^a^	85 (78, 92)	82 (76, 90)
BMI (kg/m^2^)	27.1 (24.5, 29.9)	26.2 (23.9, 28.8)
Age at recruitment (years)	56.8 (52.0, 61.9)	56.9 (51.9, 61.8)
Age at diagnosis (years)	63.8 (59.0, 68.1)	NA
Time from blood draw to diagnosis (years)	6.8 (3.3, 9.5)	NA
Folate (nmol/L)	11.5 (8.4, 17.1)	12.0 (8.6, 17.1)
SDMA (μmol/L)	0.440 (0.380, 0.500)	0.440 (0.380, 0.500)
Neopterin (nmol/L)	13.4 (10.6, 16.6)	12.7 (10.3, 15.8)

*Note*: Frequencies are shown for categorical variables. Median and IQR are shown for continuous variables.

Abbreviations: BMI, body mass index; BP, blood pressure; EPIC, European Prospective Investigation into Cancer and Nutrition; RCC, renal cell carcinoma; SDMA, symmetric dimethylarginine.

^a^ N = 367 cases and 367 controls for blood pressure measurements.

**TABLE 2:** Median and IQR of transsulfuration pathway metabolite plasma concentrations in RCC cases and matched controls at baseline in a nested case–control study within EPIC

Transsulfuration metabolite	Cases (N = 455)	Controls (N = 455)
PLP (nmol/L)	30.4 (22.3, 42.8)	36.4 (25.6, 52.0)
Total homocysteine (μmol/L)	9.41 (7.91, 11.28)	9.56 (7.71, 11.40)
Serine (μmol/L)	94.8 (82.9, 109.5)	97.7 (85.6, 112.5)
Cystathionine (μmol/L)	0.180 (0.130, 0.260)	0.180 (0.130, 0.250)
Cysteine (μmol/L)	252 (231, 277)	255 (232, 280)

Abbreviations: EPIC, European Prospective Investigation into Cancer and Nutrition; IQR, interquartile range; PLP, pyridoxal 5′‐phosphate; RCC, renal cell carcinoma.

The association between PLP and RCC risk was similar across the three models with an OR (90% CrI) for a 1 SD increase in log PLP of 0.72 (0.64, 0.82), 0.75 (0.66, 0.86) and 0.76 (0.66, 0.87) from the minimally adjusted, BMI and smoking adjusted, and mutually adjusted models, respectively (Table 3). We also found evidence of an inverse association between cysteine concentration and RCC risk (mutually adjusted OR 0.81, 90% CrI 0.66, 0.96) and an estimate for cystathionine in the direction of a positive association (mutually adjusted OR 1.12, 90% CrI 0.97, 1.30). Systolic and diastolic blood pressure were available for 367 matched pairs, and among these participants there was no notable change in the estimates for any of the five metabolites after adjustment for systolic blood pressure, diastolic blood pressure or both (Table S2).

**TABLE 3:** Odds ratios (OR) and 90% credible intervals (CrI) of transsulfuration metabolites (per 1 SD increment) with risk of RCC in a nested case‐control study in EPIC (N = 455 cases and 455 controls)

	Separate models		Combined model
	Minimally adjusted	Additionally adjusted (BMI and smoking status)	Mutually adjusted (all metabolites)
Transsulfuration metabolite	OR (90% CrI)	OR (90% CrI)	OR (90% CrI)
PLP	0.72 (0.64, 0.82)	0.75 (0.66, 0.86)	0.76 (0.66, 0.87)
Homocysteine	1.07 (0.94, 1.22)	1.04 (0.92, 1.19)	1.10 (0.92, 1.32)
Serine	0.85 (0.75, 0.96)	0.89 (0.78, 1.00)	0.90 (0.79, 1.04)
Cystathionine	1.16 (1.02, 1.33)	1.12 (0.98, 1.29)	1.12 (0.97, 1.30)
Cysteine	0.87 (0.76, 1.00)	0.84 (0.73, 0.97)	0.81 (0.66, 0.96)

*Note*: Matching variables are country, sex, age and date of blood draw. Model covariates are as follows: Minimally adjusted: education level (four categories) and fasting status (yes, in between, no). Additionally adjusted for BMI and smoking status: Minimally adjusted plus BMI (continuous) and smoking status (never, former, current). Mutually adjusted for all metabolites: All previously listed covariates plus folate concentration and all five metabolites (continuous, per 1 SD of log transformed concentration). Assessed by Bayesian conditional logistic regression, conditioning on individual case sets.

Abbreviations: BMI, body mass index; CrI, credible interval; EPIC, European Prospective Investigation into Cancer and Nutrition; OR, odds ratio; PLP, pyridoxal 5′‐phosphate; RCC, renal cell carcinoma.

RCC risk was greater for the low PLP metabolite profile compared to the high PLP profile (OR 1.88, 90% CrI 1.44, 2.49). An even stronger association was seen for the profile representative of low transsulfuration function compared to high function (OR 2.70, 90% CrI 1.26, 5.70) (Table 4). The estimated association for low vs high CSE function (OR 3.40, 90% CrI 1.99, 5.91) was stronger than that for CBS function (OR 1.37, 90% CrI 0.73, 2.60).

**TABLE 4:** Odds ratios and 90% credible intervals of transsulfuration metabolite profiles with risk of RCC in a nested case‐control study in EPIC (N = 455 cases and 455 controls)

Basis of comparison	Profile	OR (90% CrI)
Expected metabolite concentrations at specified PLP concentration^a^	High	Reference
	Moderate	1.37 (1.20, 1.58)
	Low	1.88 (1.44, 2.49)
Theoretically expected metabolite concentrations at differing levels of transsulfuration function^b^	High	Reference
	Moderate	1.64 (1.12, 2.39)
	Low	2.70 (1.26, 5.70)
Theoretically expected metabolite concentrations at differing levels of CBS function^c^	High	Reference
	Moderate	1.17 (0.85, 1.61)
	Low	1.37 (0.73, 2.60)
Theoretically expected metabolite concentrations at differing levels of CSE function^d^	High	Reference
	Moderate	1.85 (1.41, 2.43)
	Low	3.40 (1.99, 5.91)

*Note*: Profiles are joint estimates from the mutually adjusted model.

Abbreviations: CBS, cystathionine β‐synthase; CrI, credible interval; CSE, cystathionine γ‐lyase; EPIC, European Prospective Investigation into Cancer and Nutrition; OR, odds ratio; PLP, pyridoxal 5′‐phosphate; RCC, renal cell carcinoma.

^a^ PLP is set to 1, 0 and −1 SD from the mean for high, mid and low profiles. Other biomarkers were set to their conditional expected values based on linear regression models of the log concentration of each biomarker on log PLP.

^b^ Metabolite values (SD from the mean) for high, mid and low profiles, respectively: PLP 1, 0, −1; homocysteine −1, 0, 1; serine −1, 0, 1; cystathionine 0, 0, 0; cysteine 1, 0, −1.

^c^ Metabolite values (SD from the mean) for high, mid and low profiles, respectively: PLP 1, 0, −1; homocysteine −1, 0, 1; serine −1, 0, 1; cystathionine 1, 0, −1.

^d^ Metabolite values (SD from the mean) for high, mid and low profiles, respectively: PLP 1, 0, −1; cystathionine −1, 0, 1; cysteine 1, 0, −1.

**FIGURE 1:**
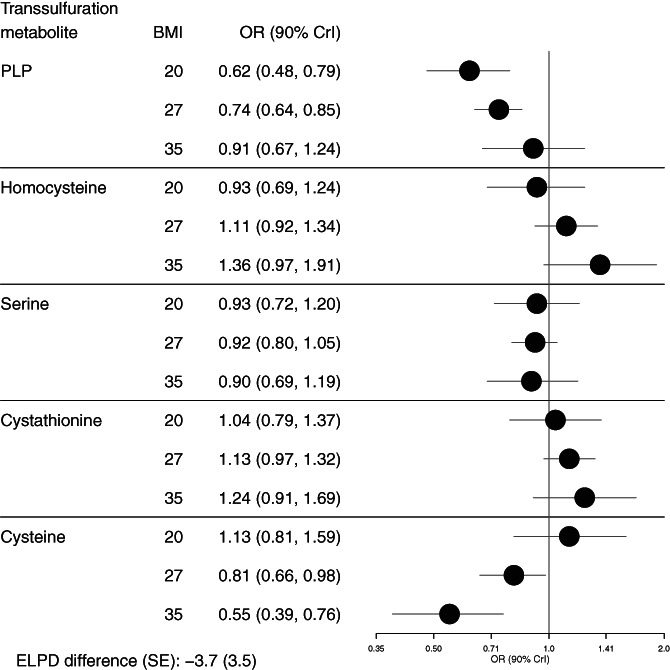
Associations of transsulfuration metabolites with risk of RCC at specified levels of BMI within a nested case‐control study in EPIC (N = 455 cases and 455 controls). Estimates shown are contrasts from the mutually adjusted model with separate interaction terms added for each metabolite with BMI as a continuous predictor. The expected log predictive density (ELPD) difference and its SE were used for model comparison against the mutually adjusted model without interaction terms. A negative ELPD indicates a worse fit for the interaction model, and the SE indicates the precision of the comparison of model fit

**FIGURE 2:**
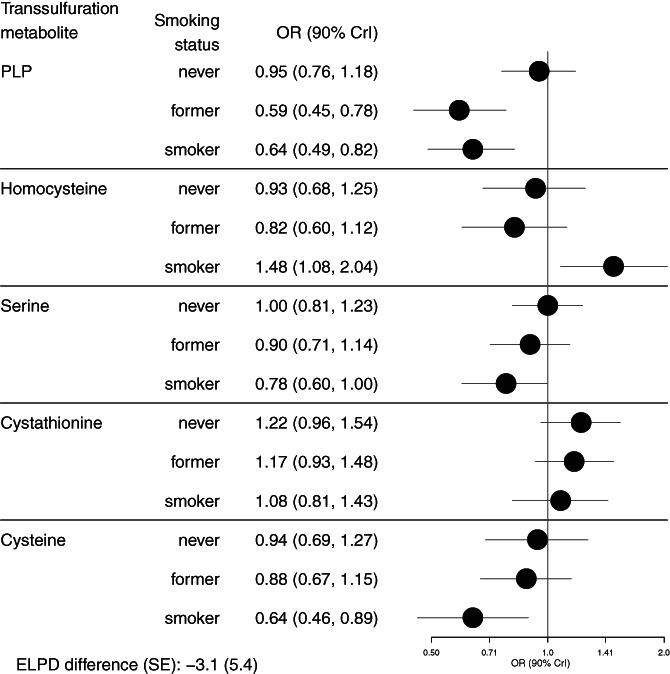
Associations of transsulfuration metabolites with risk of RCC at specified levels of smoking status within a nested case‐control study in EPIC (N = 455 cases and 455 controls). Estimates shown are contrasts from the mutually adjusted model with separate interaction terms added for each metabolite with smoking status. The expected log predictive density (ELPD) difference and its SE were used for model comparison against the mutually adjusted model without interaction terms. A negative ELPD indicates a worse fit for the interaction model, and the SE indicates the precision of the comparison of model fit

There were some variations in the strengths of associations of the transsulfuration metabolites with RCC at different levels of established RCC risk factors. We found statistical evidence of a stronger association for cysteine with RCC risk at higher compared to lower levels of BMI, with an OR of 0.55 (90% CrI 0.39, 0.76) at a BMI of 35 kg/m^2^ (Figure 1, Table S3). We also found statistical evidence of interactions with smoking status, with stronger associations of both PLP (inversely) and homocysteine (positively) with RCC risk among current smokers compared to never smokers (Figure 2, Table S3). There were no notable interactions with sex. There was evidence of an interaction of serine with systolic and diastolic blood pressure with inverse associations among those with lower blood pressure, and an interaction of PLP with systolic blood pressure with a stronger inverse association among those with lower systolic blood pressure (Figures S3‐S5 and Table S3).

Adjustment for SDMA (kidney function) and neopterin (cellular immune activation) did not notably change any reported metabolite associations with RCC risk (Table S4). Results were also similar after removing circulating folate from the fully adjusted model.

## 4 DISCUSSION

We found that multiple components of the transsulfuration pathway were associated with risk of RCC. The strength and direction of some of these associations depended on adjustment for other metabolites as well as interactions with established RCC risk factors. The inverse association of PLP with RCC risk was not attenuated by adjustment for BMI, smoking status or other transsulfuration metabolites. The strong association we found for metabolite profiles of regulation of overall transsulfuration activity, with over 2‐fold differences in risk for low compared to high function, indicates that the transsulfuration pathway may play a role in RCC etiology beyond any individual role of PLP. Furthermore, the strong association found for CSE profiles suggests the second half of the transsulfuration pathway may be the driver behind any overall association with RCC risk.

Although we did not find strong statistical evidence of interactions overall, we did observe an indication that the inverse association between cysteine and RCC risk became stronger with higher BMI. The relationship between cysteine and BMI is complex, and while a strong positive association between them has been established, the causal direction of the association is unclear.^20^ Evidence from the Hordaland Homocysteine Study suggests the association between cysteine and BMI is driven by its strong association with fat mass. This association with fat mass was independent of diet and exercise as well as cholesterol concentration. There was no evidence of a link between cysteine and lean mass independent of fat mass.^20^ Further investigation of the role of body composition may help to explain the relationship between cysteine and BMI with cancer risk.

We also found evidence of stronger associations for PLP and homocysteine with RCC risk among current smokers compared to never smokers. Because cigarette smoking is responsible for increased production of reactive oxygen species and heightened inflammatory processes,^21^ it is possible that smokers have a higher requirement for transsulfuration function to deal with these biological stressors.

Based on both empirically and theoretically constructed joint metabolite profiles, we found strong evidence of associations between transsulfuration pathway‐wide variation and RCC risk. Oxidative stress response is a possible mechanistic explanation for this finding. In a state of high oxidative stress, PLP and cysteine are likely decreased, and homocysteine is increased,^5^, ^22^, ^23^ which aligns with the directions of variation in metabolite concentrations used to build the transsulfuration function profiles. Transsulfuration is critical to oxidative stress response because the synthesis of glutathione, a free radical scavenger, is dependent on cysteine.^24^ Accumulation of reactive oxygen species results in decreased flux of one‐carbon units through the methionine cycle in favor of an increase is transsulfuration activity.^24^ Our finding that CSE function appears to be more strongly associated with RCC risk than CBS function may be explained by a greater loss of activity for CSE compared to CBS in the presence of PLP insufficiency.^25^

There is an established relationship between vitamin B6 and cancer risk. A meta‐analysis found an inverse association between PLP concentration and risk of all cancers, with 34% lower risk among participants in the highest vs lowest category.^26^ The associations of other components of transsulfuration with cancer risk are less clear. In the Northern Sweden Health and Disease Study, a Bayesian network analysis of one‐carbon metabolism, including transsulfuration metabolites as well as other folate and methionine cycle metabolites, identified an independent association of circulating PLP with colorectal cancer risk, but no direct associations for homocysteine, serine, cystathionine or cysteine.^27^ However, in the same cohort, a positive association was found between the ratio homocysteine:cysteine and colorectal cancer risk.^28^ A 43% lower colorectal cancer risk was found in a comparison of highest to lowest fourths of cysteine concentration in the Women's Health Initiative cohort.^29^ A case‐cohort study in Linxian, China found an inverse association for circulating cysteine with both gastric and esophageal cancers.^30^ Cystathionine and CBS are both found in higher concentration in breast cancer tumor tissue compared to healthy breast tissue,^31^ and an analysis from the Women's Health Study found a positive association between circulating cysteine and breast cancer risk.^32^ Findings from meta‐analyses have shown higher concentrations of homocysteine among lung cancer cases compared to controls,^33^ but no evidence of an association between homocysteine concentration and prostate cancer risk.^34^ A nested case‐control study using four US cohorts found no associations between plasma PLP or homocysteine and pancreatic cancer risk. However, the risk estimates were in the same direction as those we found for RCC risk (OR above 1 for homocysteine and below 1 for PLP).^35^

Strengths of our analysis include the use of a multinational study representing a wide range of dietary preferences and lifestyle differences. Because this case‐control study was nested within the EPIC cohort, plasma samples were collected at baseline in all participants, with data available for cases often years prior to their diagnosis. This allowed us to minimize the risk of reverse causation. Additionally, samples for all participants were analyzed at the same lab under the same protocol, and at the same time, minimizing the risk of measurement errors.

There were limitations of this investigation as well. Residual confounding is possible, in part because we did not have data on germline genetic mutations for our cases or controls, and therefore could not account for differences in *CBS* or other transsulfuration pathway genes. Additionally, the function of CBS is strongly influenced by allosteric regulation by SAM, which was not measured in our study. We also did not have data for downstream products of transsulfuration, namely glutathione. We had a limited sample size with only one control matched to each case. Metabolites were measured in blood samples taken at a single timepoint, so we could not determine the intrapersonal stability of these markers or assess associations of trajectories in transsulfuration biomarkers over time with RCC risk. However, an analysis of biomarkers measured at the same lab (Bevital) comparing samples drawn at different time points found good within‐person reproducibility for all metabolites used in our analysis, with cysteine and serine having the highest intraclass correlation coefficients.^36^

Regarding the joint metabolite profile comparisons, it should be noted that only the directions, but not the magnitudes, of assigned values (except for empirically determined values in the PLP profiles) have a strong theoretical basis supported by the literature. While our approach assumes equal variation for all metabolites, in reality there is likely a different strength of association for different cofactors, substrates and products with regulatory control of enzyme function. Therefore, our results should be considered a preliminary attempt at quantifying the function of a complex pathway.

In summary, we identified individual metabolite and pathway‐wide associations linking homocysteine metabolism via transsulfuration to RCC risk. While recent years have seen an increasing interest in the role of the folate and methionine cycles in carcinogenesis, the closely related transsulfuration pathway has received relatively little attention, but may play a central role because of its involvement in several key metabolic processes including oxidative stress response.

## CONFLICT OF INTEREST

The authors report no potential conflicts of interest.

## AUTHOR CONTRIBUTIONS

Joanna L. Clasen, Alicia K. Heath and David C. Muller were responsible for conceptualization and methodology. Joanna L. Clasen conducted the formal analysis, visualization and writing—original draft. All authors contributed to writing—review & editing. David C. Muller was responsible for supervision. The work reported in the study has been performed by the authors, unless clearly specified in the text.

## ETHICS STATEMENT

Ethics approval for the study was obtained from the International Agency for Research on Cancer and the local review boards at the participating centers. All EPIC participants provided written informed consent at baseline for use of their blood samples and data in future research.

## Supporting information


**Appendix S1** Supporting Information.

## Data Availability

For information on how to submit an application for gaining access to EPIC data and/or biospecimens, please follow the instructions at http://epic.iarc.fr/access/index. Further details and other data that support the findings of our study are available from the corresponding author upon request.
