# Supplementary material for: Biomarkers of the transsulfuration pathway and risk of renal cell carcinoma in the European Prospective Investigation into Cancer and Nutrition (EPIC) study
Source: Int J Cancer. 2022 Apr 16;151(5):708–16. doi: 10.1002/ijc.34009 (PMC9545591; doi:10.1002/ijc.34009)
Supplement: Supplementary file 1 — Appendix S1 Supporting Information. [file IJC-151-708-s001.pdf]

Biomarkers of the transsulfuration pathway and risk of renal cell carcinoma in the European Prospective Investigation into Cancer and Nutrition (EPIC) study

Joanna L Clasen; Alicia K Heath; Heleen Van Puyvelde; Inge Huybrechts; Jin Young Park; Pietro Ferrari; Ghislaine Scelo; Arve Ulvik; Øivind Midttun; Per Magne Ueland; Kim Overvad; Anne Kirstine Eriksen; Anne Tjønneland; Rudolf Kaaks; Verena Katzke; Matthias B Schulze; Domenico Palli; Claudia Agnoli; Paolo Chiodini; Rosario Tumino; Carlotta Sacerdote; Raul Zamora-Ros; Miguel Rodriguez-Barranco; Carmen Santiuste; Eva Ardanaz; Pilar Amiano; Julie A Schmidt; Elisabete Weiderpass; Marc Gunter; Elio Riboli; Amanda J Cross; Mattias Johansson; David C Muller

Table of Contents

Supplementary Tables 2

Supplementary Figures 8

Supplementary Tables

Supplementary Table 1: Participants from the EPIC RCC nested case-control study included and excluded from the analysis of transsulfuration metabolites and RCC risk. Participants were excluded if they were missing any biomarker or covariate data.

	**Excluded (N=194)**	**Included (N=910)**
Sex, N (%)		
Male	96 (49%)	512 (56%)
Female	98 (51%)	398 (44%)
Smoking status, N (%)		
Never	98 (53%)	371 (41%)
Former	53 (28%)	286 (31%)
Current	35 (19%)	253 (28%)
Age at recruitment (years), median (range)	57.6 (39.9, 74.7)	56.8 (36.0, 74.1)
BMI (kg/m^2), median (range)	25.4 (18.6, 43.1)	26.5 (14.7, 47.2)

Abbreviations: body mass index (BMI), European Prospective Investigation into Cancer and Nutrition (EPIC), renal cell carcinoma (RCC)

Supplementary Table 2: Odds ratios and 90% credible intervals of transsulfuration metabolites (per 1 SD increment) with risk of RCC among participants with measured blood pressure in a nested case-control study in EPIC (N=734).

Transsulfuration metabolite	Not adjusted for blood pressure	Adjusted for systolic blood pressure	Adjusted for diastolic blood pressure	Adjusted for systolic and diastolic blood pressure
PLP	0.76 (0.64, 0.88)	0.78 (0.67, 0.91)	0.77 (0.66, 0.90)	0.78 (0.67, 0.91)
Homocysteine	1.22 (0.99, 1.52)	1.21 (0.99, 1.51)	1.20 (0.98, 1.49)	1.22 (0.98, 1.51)
Serine	0.93 (0.80, 1.08)	0.95 (0.81, 1.10)	0.94 (0.81, 1.09)	0.95 (0.81, 1.10)
Cystathionine	1.13 (0.96, 1.35)	1.16 (0.98, 1.39)	1.14 (0.96, 1.36)	1.17 (0.98, 1.40)
Cysteine	0.72 (0.58, 0.89)	0.68 (0.54, 0.85)	0.70 (0.56, 0.87)	0.68 (0.54, 0.85)

Matching variables are country, sex, age, and date of blood draw.

All models adjusted for education level (four categories), fasting status (yes, in between, no), BMI (continuous), smoking status (never, former, current), folate concentration and the five metabolites (continuous, per 1 SD of log transformed concentration), and blood pressure as shown in each column.

Assessed by Bayesian conditional logistic regression, conditioning on individual case sets.

Abbreviations: body mass index (BMI), European Prospective Investigation into Cancer and Nutrition (EPIC), pyridoxal 5'-phosphate (PLP), renal cell carcinoma (RCC), standard deviation (SD)

Supplementary Table 3: Odds ratios and 90% credible intervals of transsulfuration metabolites (per 1 SD increment) with risk of RCC and their interactions with established RCC risk factors (per 1 SD increment for continuous variable risk factors) in a nested case-control study in EPIC.

	Interactions with BMI	Interactions with smoking status[1]	Interactions with sex[2]	Interactions with systolic BP	Interactions with diastolic BP
PLP	0.74 (0.64, 0.86)	0.95 (0.76, 1.18)	0.67 (0.53, 0.82)	0.79 (0.67, 0.93)	0.79 (0.67, 0.93)
Homocysteine	1.11 (0.93, 1.34)	0.93 (0.68, 1.25)	1.14 (0.90, 1.45)	1.25 (1.00, 1.56)	1.22 (1.00, 1.52)
Serine	0.92 (0.80, 1.05)	1.00 (0.81, 1.23)	0.90 (0.74, 1.08)	0.93 (0.80, 1.09)	0.96 (0.83, 1.12)
Cystathionine	1.13 (0.97, 1.32)	1.22 (0.96, 1.54)	1.12 (0.92, 1.36)	1.19 (0.99, 1.44)	1.18 (0.98, 1.40)
Cysteine	0.80 (0.66, 0.97)	0.94 (0.69, 1.27)	0.75 (0.58, 0.95)	0.65 (0.52, 0.82)	0.67 (0.54, 0.85)
BMI x PLP	1.12 (0.98, 1.29)				
BMI x homocysteine	1.11 (0.96, 1.29)				
BMI x serine	0.99 (0.87, 1.13)				
BMI x cystathionine	1.05 (0.91, 1.21)				
BMI x cysteine	0.81 (0.69, 0.95)				
Smoking status x PLP					
Never		reference			
Former		0.62 (0.44, 0.89)			
Current		0.68 (0.49, 0.95)			
Smoking status x homocysteine					
Never		reference			
Former		0.89 (0.59, 1.34)			
Current		1.60 (1.04, 2.43)			
Smoking status x serine					
Never		reference			
Former		0.90 (0.66, 1.24)			
Current		0.78 (0.57, 1.07)			
Smoking status x cystathionine					
Never		reference			

	Interactions with BMI	Interactions with smoking status[1]	Interactions with sex[2]	Interactions with systolic BP	Interactions with diastolic BP
Former		0.97 (0.70, 1.31)			
Current		0.89 (0.63, 1.25)			
Smoking status x cysteine					
Never		reference			
Former		0.93 (0.65, 1.34)			
Current		0.68 (0.45, 1.03)			
Sex x PLP					
Men			reference		
Women			1.26 (0.94, 1.68)		
Sex x homocysteine					
Men			reference		
Women			0.93 (0.64, 1.34)		
Sex x serine					
Men			reference		
Women			1.04 (0.80, 1.36)		
Sex x cystathionine					
Men			reference		
Women			1.00 (0.74, 1.36)		
Sex x cysteine					
Men			reference		
Women			1.17 (0.82, 1.68)		
Systolic BP x PLP				1.19 (1.02, 1.39)	
Systolic BP x homocysteine				1.09 (0.91, 1.31)	
Systolic BP x serine				1.25 (1.08, 1.44)	
Systolic BP x cystathionine				1.03 (0.88, 1.23)	

	Interactions with BMI	Interactions with smoking status[1]	Interactions with sex[2]	Interactions with systolic BP	Interactions with diastolic BP
Systolic BP x cysteine				0.94 (0.78, 1.14)	
Diastolic BP x PLP					1.04 (0.89, 1.23)
Diastolic BP x homocysteine					1.10 (0.93, 1.33)
Diastolic BP x serine					1.22 (1.04, 1.44)
Diastolic BP x cystathionine					1.05 (0.90, 1.23)
Diastolic BP x cysteine					0.89 (0.75, 1.06)

Matching variables are country, sex, age, and date of blood draw.

Adjusted for education level (four categories), fasting status (yes, in between, no), BMI (continuous), smoking status (never, former, current), folate concentration and the five metabolites (continuous, per 1 SD of log transformed concentration).

Sex interaction model does not include main effects estimates for sex.

Systolic and diastolic BP are adjusted for respectively in their interaction models, and models are fit in the subset of participants (N=734).

Assessed by Bayesian conditional logistic regression, conditioning on individual case sets.

Abbreviations: body mass index (BMI), blood pressure (BP), European Prospective Investigation into Cancer and Nutrition (EPIC), pyridoxal 5'-phosphate (PLP), renal cell carcinoma (RCC), standard deviation (SD)

[1]Metabolite main effects represent estimates for never smokers

[2]Metabolite main effects represent estimates for men

Supplementary Table 4: Odds ratios and 90% credible intervals from sensitivity analyses of transsulfuration metabolites (per 1 SD increment) with risk of RCC in a nested case-control study in EPIC. Adjusted for markers of kidney function (SDMA) and immune activation (neopterin) (N=908) and without adjusting for circulating folate (N=910).

Transsulfuration metabolite	Adjusted for SDMA and neopterin OR (90% CrI)[1]	Not adjusted for folate OR (90% CrI)[2]
PLP	0.76 (0.65, 0.87)	0.77 (0.68, 0.89)
Homocysteine	1.11 (0.93, 1.34)	1.05 (0.90, 1.24)
Serine	0.92 (0.80, 1.05)	0.90 (0.79, 1.03)
Cystathionine	1.14 (0.98, 1.33)	1.11 (0.96, 1.28)
Cysteine	0.81 (0.68, 0.98)	0.84 (0.71, 0.99)

[1]Adjusted for education level (four categories), fasting status (yes, in between, no), BMI (continuous), smoking status (never, former, current), folate concentration and the five transsulfuration metabolites, SDMA, and neopterin (continuous, per 1 SD of log transformed concentration).

[2]Adjusted for education level (four categories), fasting status (yes, in between, no), BMI (continuous), smoking status (never, former, current), and the five transsulfuration metabolites.
Matching variables are country, sex, age, and date of blood draw.
Assessed by Bayesian conditional logistic regression, conditioning on individual case sets.
Abbreviations: body mass index (BMI), European Prospective Investigation into Cancer and Nutrition (EPIC), pyridoxal 5'-phosphate (PLP), renal cell carcinoma (RCC), standard deviation (SD), symmetric dimethylarginine (SDMA)

Supplementary Figures

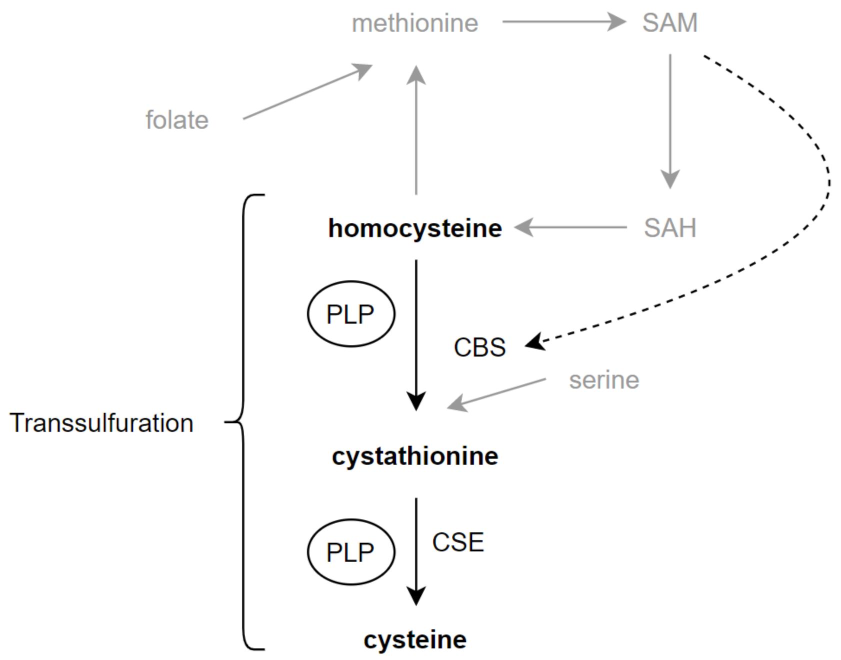

Supplementary Figure 1: Key metabolites, enzymes, and cofactors of the transsulfuration pathway and the methionine cycle. Homocysteine and serine are condensed by the enzyme CBS to form cystathionine. Cystathionine is converted to cysteine by the enzyme CSE. PLP is a cofactor for both enzymes. Allosteric activation by SAM is indicated by the dashed arrow. Abbreviations: cystathionine β-synthase (CBS), cystathionine γ-lyase (CSE), pyridoxal 5’-phosphate (PLP), S-adenosylhomocysteine (SAH), S-adenosylmethionine (SAM).

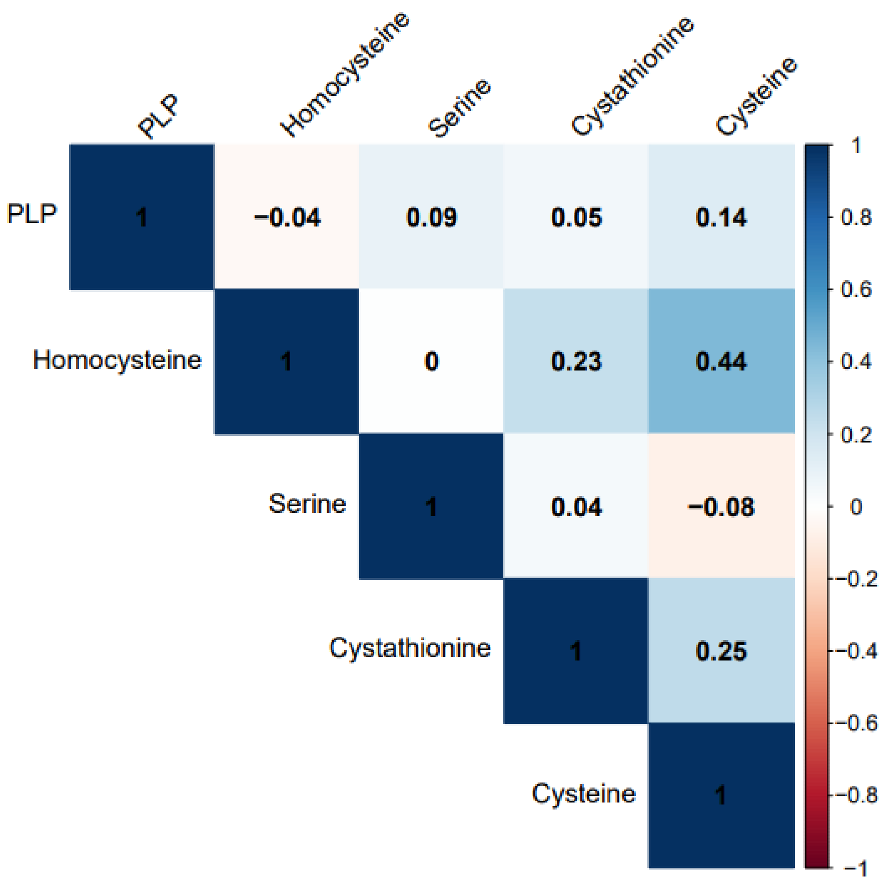

Supplementary Figure 2: Correlations between transsulfuration metabolites in controls from a nested case-control study in EPIC (N=455) using the Pearson correlation coefficient.

Transsulfuration metabolite	Sex	OR (90% CrI)
PLP	men	0.67 (0.53, 0.82)
	women	0.83 (0.69, 1.00)
Homocysteine	men	1.14 (0.90, 1.45)
	women	1.05 (0.79, 1.41)
Serine	men	0.90 (0.74, 1.08)
	women	0.93 (0.77, 1.12)
Cystathionine	men	1.12 (0.92, 1.36)
	women	1.12 (0.88, 1.42)
Cysteine	men	0.75 (0.58, 0.95)
	women	0.87 (0.66, 1.15)

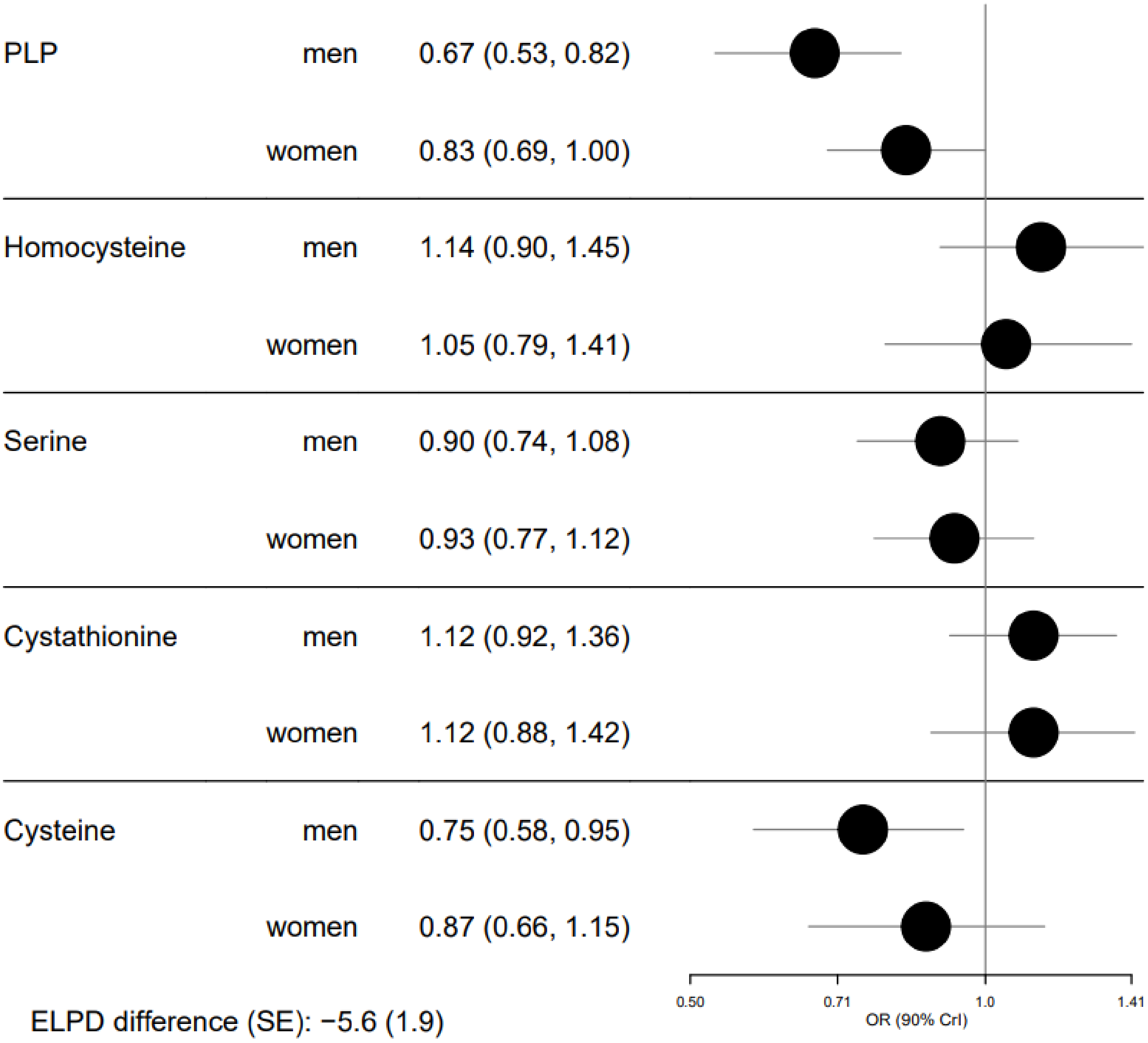

ELPD difference (SE): −5.6 (1.9)

Supplementary Figure 3: Associations of transsulfuration metabolites with risk of RCC by sex. Estimates shown are contrasts from the mutually adjusted model with separate interaction terms added for each metabolite with sex. The expected log predictive density (ELPD) difference and its standard error (SE) were used for model comparison against the mutually adjusted model without interaction terms. A negative ELPD indicates a worse fit for the interaction model, and the SE indicates the precision of the comparison of model fit. From a nested case-control study in EPIC (N=910).

Transsulfuration metabolite	Systolic BP	OR (90% CrI)
PLP	110	0.63 (0.47, 0.82)
	135	0.77 (0.66, 0.91)
	160	0.96 (0.76, 1.21)
Homocysteine	110	1.12 (0.78, 1.61)
	135	1.24 (0.98, 1.56)
	160	1.37 (1.05, 1.84)
Serine	110	0.70 (0.54, 0.91)
	135	0.91 (0.78, 1.07)
	160	1.19 (0.96, 1.48)
Cystathionine	110	1.14 (0.87, 1.50)
	135	1.19 (0.99, 1.43)
	160	1.24 (0.94, 1.66)
Cysteine	110	0.71 (0.50, 1.00)
	135	0.66 (0.52, 0.83)
	160	0.61 (0.45, 0.83)

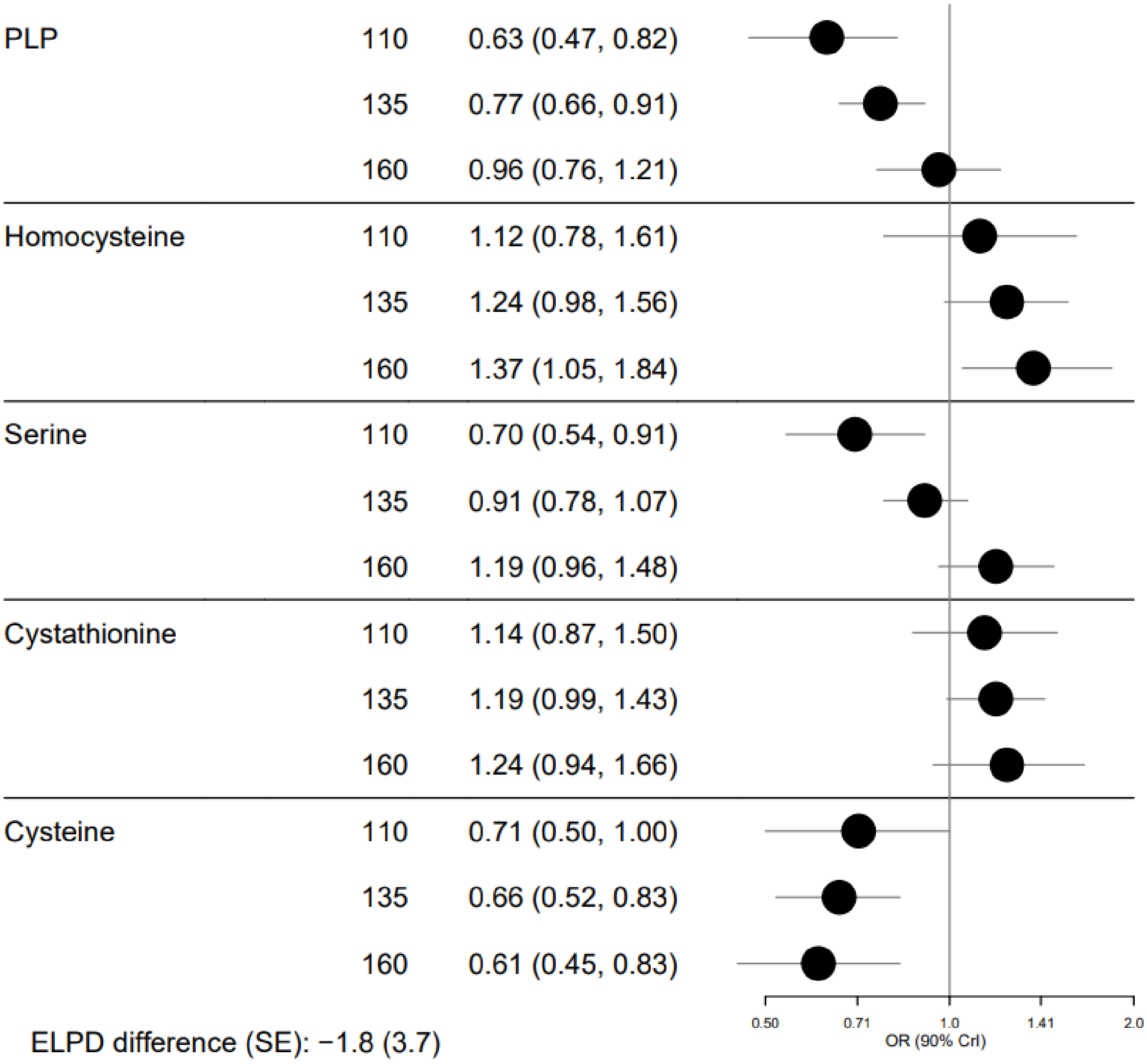

ELPD difference (SE): −1.8 (3.7)

Supplementary Figure 4: Associations of transsulfuration metabolites with risk of RCC at specified levels of systolic blood pressure (BP). Estimates shown are contrasts from the mutually adjusted model with separate interaction terms added for each metabolite with systolic BP as a continuous predictor. The expected log predictive density (ELPD) difference and its standard error (SE) were used for model comparison against the mutually adjusted model without interaction terms. A negative ELPD indicates a worse fit for the interaction model, and the SE indicates the precision of the comparison of model fit. From a nested case-control study in EPIC (N=734).

Transsulfuration metabolite	Diastolic BP	OR (90% CrI)
PLP	70	0.75 (0.59, 0.94)
	85	0.79 (0.67, 0.93)
	100	0.84 (0.63, 1.11)
Homocysteine	70	1.08 (0.80, 1.46)
	85	1.23 (1.01, 1.53)
	100	1.40 (1.04, 1.92)
Serine	70	0.76 (0.59, 0.97)
	85	0.98 (0.84, 1.14)
	100	1.27 (0.97, 1.66)
Cystathionine	70	1.11 (0.88, 1.41)
	85	1.18 (0.99, 1.41)
	100	1.25 (0.94, 1.70)
Cysteine	70	0.77 (0.58, 1.03)
	85	0.67 (0.53, 0.84)
	100	0.57 (0.40, 0.82)

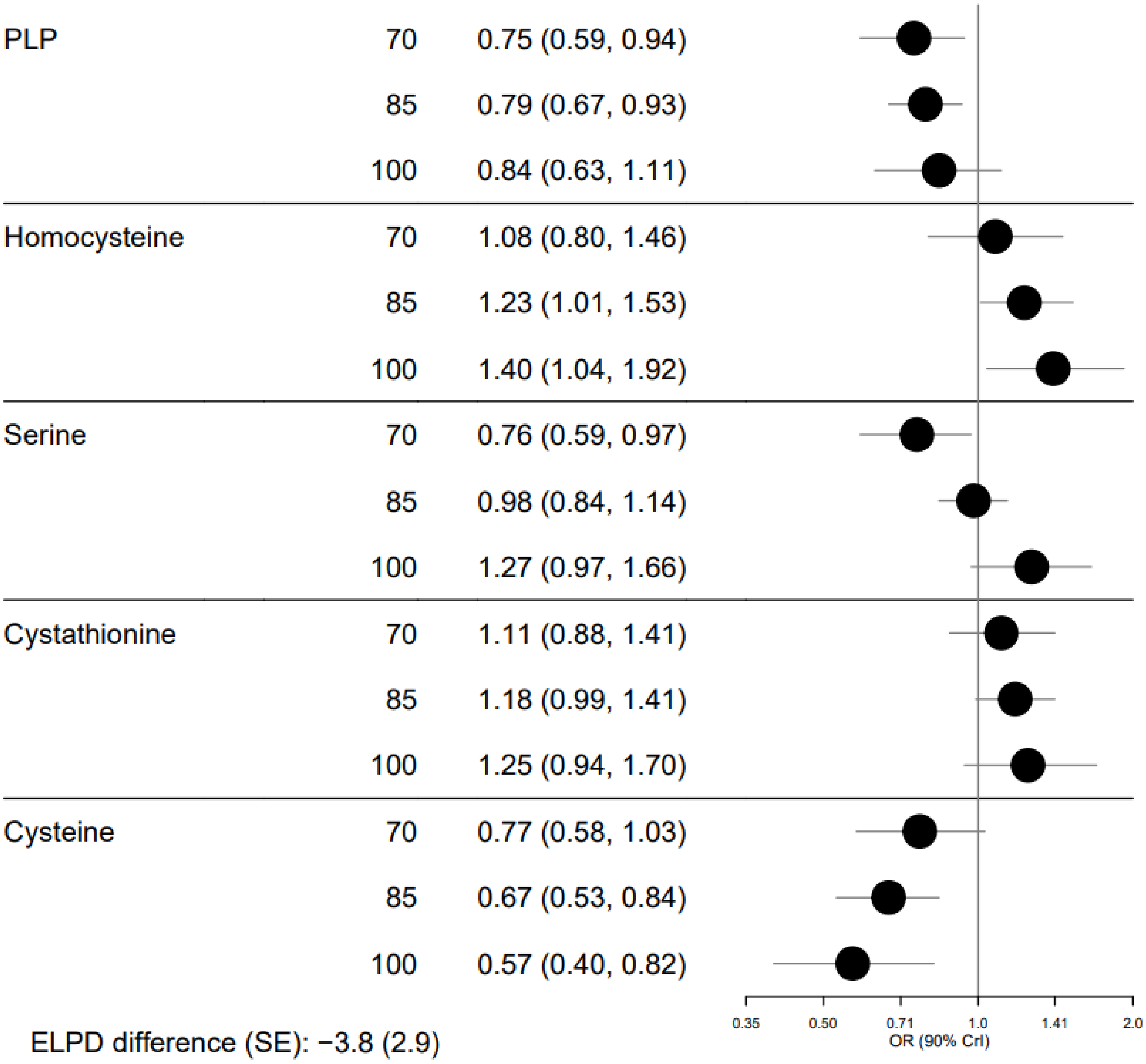

ELPD difference (SE): −3.8 (2.9)

Supplementary Figure 5: Associations of transsulfuration metabolites with risk of RCC at specified levels of diastolic blood pressure (BP). Estimates shown are contrasts from the mutually adjusted model with separate interaction terms added for each metabolite with diastolic BP as a continuous predictor. The expected log predictive density (ELPD) difference and its standard error (SE) were used for model comparison against the mutually adjusted model without interaction terms. A negative ELPD indicates a worse fit for the interaction model, and the SE indicates the precision of the comparison of model fit. From a nested case-control study in EPIC (N=734).
